# Dengue Virus Strikes Back: Increased Future Risk of Severe Dengue Disease in Humans as a Result of Previous Exposure to Zika Virus

**DOI:** 10.3390/jcm9124060

**Published:** 2020-12-16

**Authors:** Miguel A. Martín-Acebes, Juan-Carlos Saiz, Nereida Jiménez de Oya

**Affiliations:** Department of Biotechnology, Instituto Nacional de Investigación y Tecnología Agraria y Alimentaria (INIA), 28040 Madrid, Spain; jcsaiz@inia.es

Mosquito-borne flaviviruses include medically important pathogens that are responsible for a variety of human diseases, such as dengue, Zika congenital syndrome, and West Nile fever. These pathogens are characterized by close antigenic relationships among different species, and the cross-reactivity of the host immune response is a hallmark of this group. Although this cross-reactivity may impart a certain degree of cross-protective immunity to different flaviviruses, it has also been associated with immunopathology and exacerbation of infections, with dengue virus (DENV) as a paradigm for this effect [1]. The exacerbation of infection is thought to occur through antibody-dependent enhancement (ADE): antibodies with sub-neutralizing activity can enhance viral replication in permissive cells that bear Fc receptors, leading to the ADE phenomenon [2]. Currently, dengue is endemic in over 100 countries and responsible for hundreds of thousands of clinically relevant cases with more than 20,000 annual deaths. Because pre-existing immunity to the virus can exacerbate subsequent infections with heterologous serotypes, it is a major factor for disease severity [3]. 

During the 2015–2016 Zika virus (ZIKV) epidemic, the coexistence of related flaviviruses, especially DENV, in areas with outbreaks raised the question of whether some pathological conditions might be related to the antigenic similarities among circulating flaviviruses. This hypothesis arose primarily from previous experience with dengue, as pre-existing immunity against one of its four heterologous serotypes (DENV-1–4) was observed to exacerbate subsequent infection with another serotype. Thus, the emergence of a new antigenically related flavivirus, such as ZIKV, in areas where DENV is endemic can have unpredicted consequences. Therefore, the potential risk associated with the emergence of ZIKV in such areas is an important concern, not only for naturally occurring infections but also for potential future vaccine campaigns against these pathogens. In fact, the existence of ADE has been a major barrier to the development of safe and effective vaccines against DENV [4].

Research that addresses the potential implications of ZIKV cross-reactivity with DENV and other related flaviviruses rapidly proliferated after the explosion of ZIKV cases across the American continents. However, these studies had mixed results and, in some cases, reached contradictory conclusions. Some evidence pointed to potential cross-protection between ZIKV and other flaviviruses, whereas other findings suggested that infection was exacerbated by this cross-reactivity [5]. The inconsistent results of these studies, primarily based on in vitro and animal model data, highlight the importance of conducting seroepidemiological studies to understand the pathological conditions that naturally occur in the host during viral infections. 

To shed new light on the consequences of sequential ZIKV and DENV infections, a recent outstanding study analyzed a prospective pediatric cohort of around 3800 individuals in Nicaragua [6] who had been followed since 2004. Because these children lived in areas that experienced DENV outbreaks after the ZIKV epidemic, they were valuable study subjects. Seroepidemiological data derived from the analysis of this cohort indicated that a previous immune response against ZIKV was a risk factor for the development of severe dengue disease in the future, similar to the exacerbation of ZIKV by a prior DENV infection. Remarkably, the risk of developing severe disease from a secondary infection with DENV-2 was the same when prior immunity existed to ZIKV or DENV. However, the antigenic relationships that occur during secondary DENV infections in the presence of prior immunity to ZIKV or DENV markedly differ depending on the infecting DENV serotype, thus further complicating the picture. In addition to DENV-2, the risk of developing severe disease also occurs with DENV-3 when there is previous immunity to both ZIKV and DENV, in contrast to cases of, for example, DENV-1. Importantly, and contrary to the result of two sequential infections of DENV, prior infection with DENV followed by ZIKV increases the risk of future dengue disease. Moreover, previous immunity against both ZIKV or DENV appears to positively correlate with a reduced probability of disease in a subsequent ZIKV infection. 

What is the molecular basis behind these mixed effects? The complexity of ADE has been related to the neutralization mechanism exerted by antibodies: the ability of cross-reactive antibodies to neutralize a virus is dependent on its stoichiometry, and therefore, a correlative stoichiometry requirement is expected to also apply to ADE. Indeed, data obtained by Katzelnick and coworkers [6] suggest that there is a close relationship between antibody titers and the probability of severe disease outcomes in infected individuals with previous exposure to DENV or ZIKV. These authors identified certain ranges of antibody titers as protective or enhancers of the infection, especially for certain DENV serotypes and, although to a lesser extent, for subsequent ZIKV infection.

The evidence supporting the exacerbation of DENV infection in humans because of prior immunity against ZIKV is likely to have key implications for the understanding of the current epidemiological scenario and the future of vaccine design against these pathogens. Furthermore, the importance of antibody titers in combatting or worsening a disease also raises the possibility of a dynamic scenario that is dependent on the presence or absence of long-term immunity, acquired naturally or by vaccination. 
